# Molecular Dynamics of Retinoic Acid-Induced Craniofacial Malformations: Implications for the Origin of Gnathostome Jaws

**DOI:** 10.1371/journal.pone.0000510

**Published:** 2007-06-06

**Authors:** Maxence Vieux-Rochas, Laurent Coen, Takahiro Sato, Yukiko Kurihara, Yorick Gitton, Ottavia Barbieri, Karine Le Blay, Giorgio Merlo, Marc Ekker, Hiroki Kurihara, Philippe Janvier, Giovanni Levi

**Affiliations:** 1 Evolution des Régulations Endocriniennes, CNRS, UMR5166, Muséum National d'Histoire Naturelle, Paris, France; 2 Department of Physiological Chemistry and Metabolism, Graduate School of Medicine, University of Tokyo, Tokyo, Japan; 3 Transgenic Unit, Department of Oncology, Biology and Genetic University of Genova, Genova, Italy; 4 Dulbecco Telethon Institute CNR-ITB, Milano, Italy; 5 Centre for Advanced Research in Environmental Genomics, Department of Biology, University of Ottawa, Ottawa, Canada; 6 CNRS, UMR 5143, Département Histoire de la Terre, Muséum National d'Histoire Naturelle, Paris, France; 7 Palaeontology Department, The Natural History Museum, London, United Kingdom; Max Planck Institute of Molecular Cell Biology and Genetics, Germany

## Abstract

**Background:**

Intake of retinoic acid (RA) or of its precursor, vitamin A, during early pregnancy is associated with increased incidence of craniofacial lesions. The origin of these teratogenic effects remains enigmatic as in cranial neural crest cells (CNCCs), which largely contribute to craniofacial structures, the RA-transduction pathway is not active. Recent results suggest that RA could act on the endoderm of the first pharyngeal arch (1stPA), through a RARß-dependent mechanism.

**Methodology/Principal Findings:**

Here we show that RA provokes dramatically different craniofacial malformations when administered at slightly different developmental times within a narrow temporal interval corresponding to the colonization of the 1^st^ PA by CNCCs. We provide evidence showing that RA acts on the signalling epithelium of the 1^st^ PA, gradually reducing the expression of endothelin-1 and Fgf8. These two molecular signals are instrumental in activating *Dlx* genes in incoming CNCCs, thereby triggering the morphogenetic programs, which specify different jaw elements.

**Conclusions/Significance:**

The anatomical series induced by RA-treatments at different developmental times parallels, at least in some instances, the supposed origin of modern jaws (e.g., the fate of the incus). Our results might provide a conceptual framework for the rise of jaw morphotypes characteristic of gnathostomes.

## Introduction

Retinoic acid (RA), the active metabolite of vitamin A, is indispensable for normal morphogenesis and organogenesis of most vertebrate species. When administered during embryonic development, RA also acts as a potent teratogen in all vertebrates and even in certain invertebrates [1]–[3]. The spectrum of RA-induced malformations is highly variable depending on the dose administered and on the developmental stage at the time of exposure [1]–[3]. In humans, oral intake of RA during the first weeks of pregnancy engenders highly variable morphological and neural lesions collectively named “retinoic acid embryopathies” (RAEs); these include severe defects of the jaws and of the middle and external ear [4]–[7]. In the mouse, treatment of pregnant females or cultured embryos with RA at embryonic day (E) 8.0 induces fusion and hypoplasia of the first two pharyngeal arches (PAs). These embryonic defects could well account for the craniofacial alterations displayed at birth upon RA exposure. Indeed, most of the structures affected derive from the 1^st^ and 2^d^ PAs, which are colonized by incoming cranial neural crest cells (CNCCs) [8]–[14]. It has been repeatedly proposed that CNCCs could be the primary target of RA-induced teratogenesis of facial structures [15]–[19]. This notion derived from the fact that most dysmorphic structures in RAE are CNCC derivatives and was reinforced by initial analyses of animal models suggesting that excess RA could alter CNCC survival and migration [20], [21].

Recent studies, however, have shown that retinoid-induced fusion of the 1^st^ and 2^d^ PA occurs without any obvious alteration of CNCC migration or apoptosis [16]. More importantly, it has been shown that CNCCs colonizing the first two PAs do not express a retinoid responsive transgene upon treatment with a general agonist of RA. This finding clearly indicates that the RA-mediated transduction machinery is not active in these cells shortly after their arrival in the PAs [22]. These notion is also supported by reports showing that CNCCs, although expressing certain RA nuclear receptors [23], [24], may not respond directly to RA under physiological conditions [25]–[28]. CNCCs do not seem, therefore, to be the primary targets of RA-induced defects in derivatives of the 1^st^ PA.

Recent, grafting and fate mapping experiments have unequivocally shown that molecular signals deriving from the endodermal and ectodermal epithelial linings of the 1^st^ PA, are essential for patterning the underlying neural crest-derived ectomesenchyme [29], [30]. Indeed, the epithelial lining of the 1^st^ PA is endowed with a molecular map which conveys to CNCCs the information needed to generate craniofacial structures [29], [30]. These observations suggest that a possible target of RA activity could be the PA epithelia and that RA treatment would result in an alteration of their signals to CNCCs.

This hypothesis finds support in recent findings showing that treatment of mouse embryos with a general agonist of RA, activates ectopic RA signalling in the endoderm and ectoderm lining the first two PAs. This change in epithelial patterning is revealed by the rostral shift of the expression domains of RA-responsive genes including the *Rarehsp68-lacZ* reporter, the *RAR* ***ß*** gene, *Hoxa1* and *Hoxb1* [22]. Indeed, *Hox* expression seems to play an important role in the regionalization of the pharyngeal endoderm [28], [31], [32] a developmental function that can occur even in the absence of CNCCs [3], [33]–[35].

Taken together, these data might suggest that the craniofacial defects of RAE all derive from an abnormal signalling function of the pharyngeal epithelium to CNCCs. However, the exact mechanism underlying this action of RA, and its impact on the development and evolution of craniofacial structures remains so far unexplained.

At variance from what happens in the other PAs, CNCCs colonizing the 1^st^ PA do not express *Hox* genes [36] and their patterning depends on the expression of several families of non-*Hox* homeobox genes. In particular, *Dlx* genes, homeodomain transcription factors related to drosophila *dll*, play a pivotal role in determining mandibular and maxillar identities [37]–[40]. *Dlx1* and *Dlx2* are activated in CNCCs by Fgf8 from a signalling center in the 1^st^ PA ectoderm [41]–[44], their targeted inactivation engenders mostly maxillary arch defects with the appearance of a cartilagineous structure positionally homologous to a palatoquadrate (pq) [38]. Conversely, *Dlx5* and *Dlx6* are activated in CNCCs by ET1 signalling from the 1^st^ PA endoderm [45], [46] and their targeted inactivation leads to the transformation of the lower jaw into an upper jaw with loss of Meckel's cartilage (Mc) and the appearance, in the lower jaw, of maxillary arch derivatives such as the vibrissae and palatine rugae [39], [47]; mice lacking ET1 or its receptor A (ETRA) present a similar transformation [46], [48]. Therefore, on the basis of their nested expression pattern and of the craniofacial phenotypes of the corresponding mutant mice, it has been proposed that *Dlx* genes play a pivotal role in determining the patterning, development and evolution of vertebrate jaws [40].

In this study we explored the possibility that the craniofacial lesions present in RAE could derive from altered signalling from the PA epithelium leading to abnormal activation of *Dlx* genes in CNCCs. Our observations fully confirm this hypothesis and prompt us with new insight suggesting a possible scenario for the origin of craniofacial structures in vertebrates.

## Results

### Timing of RA teratogenic effects on craniofacial structures

We first analyzed the chondrocrania (14.5 dpc) and the dermatocrania (18.5 dpc) of mouse embryos from pregnant females that had received a single dose of RA by force-feeding (gavage). This single treatment was administered at different times within a narrow temporal window, which encompassed the period of CNCC colonization of the PAs. RA administration had the most pronounced effects on craniofacial structures in an interval ranging from 204 to 213 hours post coitum (hpc) (9 to 14 somites, corresponding to 8.5–8.75 dpc). This is the period during which CNCCs from rhombomeres 1 and 2 and the mesencephalon reach the 1^st^ PA [11]. No major defects in craniofacial structures were observed when the treatment was administered either before 202 hpc or after 215 hpc. The phenotypes obtained were variable even within a single litter, possibly due to different times of individual fertilizations and hence to the different age of individual embryos. In spite of this, by comparing the spectrum of phenotypes displayed by embryos issued from more than 40 timed-litters we were able to arrange the dysmorphologies induced by RA in an unequivocal sequential order. Here we only focus on the skeletal phenotypes, but a detailed description of other aspects of the phenotype, including an analysis of non-skeletal structures, will be presented elsewhere.

### Dynamics of chondrocranial defects induced by RA-treatment of mouse embryos

**Figure 1:**
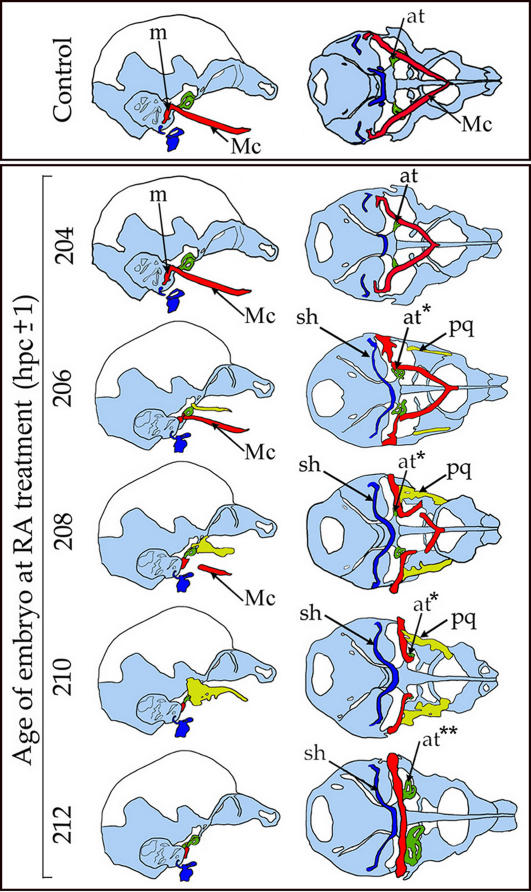
Schematic drawings representing the main features of the chondrocrania (14.5 dpc) of embryos which received a single RA pulse at the indicated developmental time (expressed in hours post coitum). Left column, lateral view; right column, caudal view. The main structures highlighted in colour are Meckel's cartilage (Mc, red), the neoformed palatoquadrate (pq, yellow), the ala temporalis (at, green), and the stylo-hyoidiean cartilage (sh). The first defect to develop is a proximal deformation of Mc with the appearance of a medially oriented cartilage bar. The malleus (m) is rapidly lost. Then the pq and the sh appear at the same time. The distal part of Mc separates, shortens progressively and eventually disappears. The pq becomes progressively more prominent, to occupy most of the maxillary region of the head. Coincidentally with the appearance of the pq, the at is duplicated (at*) and later triplicated (at**). Treatment of embryos of 211–213 hpc leads to a complete loss of Mc and of the pq.

**Figure 2:**
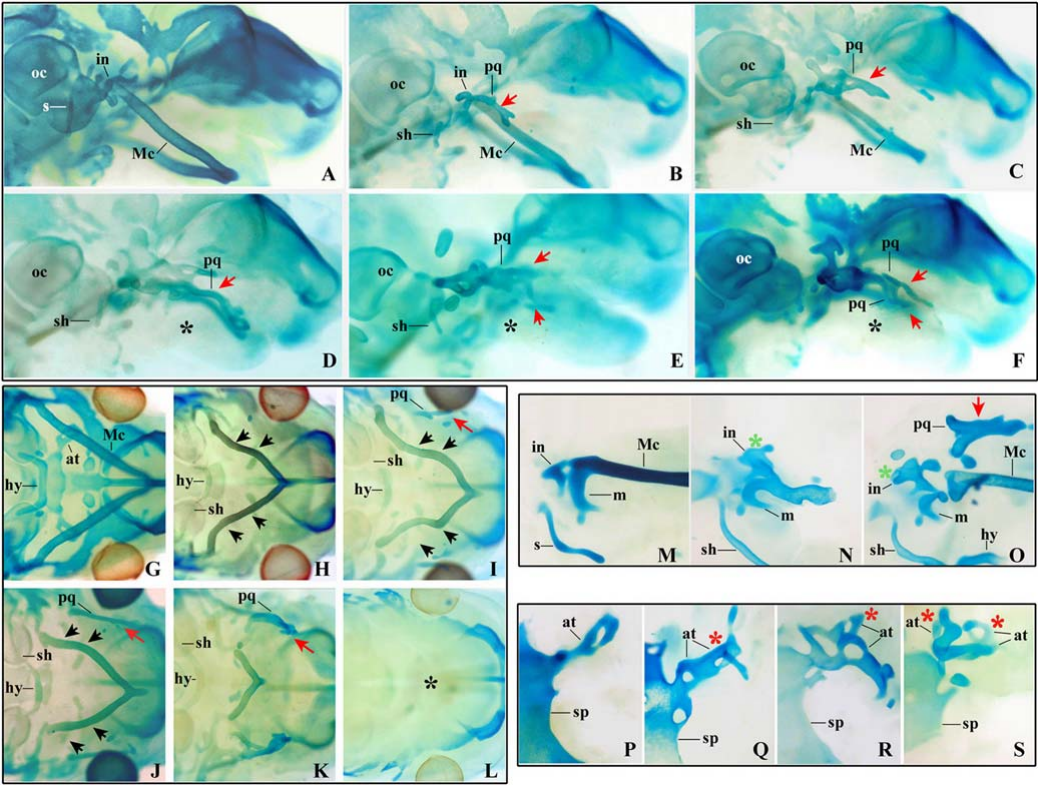
Defects of the chondrocranium induced by RA treatment at different developmental times. Lateral (A–F) or caudal (G–H) views of chondrocrania of representative 14.5 dpc embryos stained in alcian blue to reveal cartilagineous structures. The embryos derive from control mothers treated with vehicle alone (G, M, P), or from mothers exposed to a single dose of RA at 203 (A, H), 204 (B, I), 206 (C, J), 208 (K), 210 (D), 211 (E, F) and 212 (L) hpc (+/−1). Detailed views of the middle ear cartilages (M–O) and of the region of the at (P–S) in 14.5 dpc control embryos (M, P) and in embryos deriving from mothers exposed to a single dose of RA at 203 (N), 204 (O), 206 (Q), 210 (R) and 212 (S) hpc (+/−1). Early RA treatment (A, B, N) results in the progressive deformation of the incus (in, green asterisk in N, O) which elongates rostrally making initially contact with the proximal part of Meckel's cartilage (Mc) (B, N). Then the deformed incus makes contact with a new cartilage bar forming in the maxillary region (red arrow in O) and gives rise to a cartilagineous structure that occupies the same relational position as the palatoquadrate (pq) in a generalized gnathostome. Later treatments result in the formation a long flattened cartilagineous palatoquadrate in the maxillary region (D–F, J, K), this structure could be duplicated at the distalmost end (E) or appear as two parallel, highly anastomosed cartilage bars (F). Early RA treatments resulted in proximal defects of Mc with displayed a proximo median deformation (black arrows in H,I). With late treatments, Mc separates into a proximal part which generates a longitudinal bar oriented medially and a distal part which detaches, from the proximal region (J) and persists as strongly reduced distal structure (K); finally, Mc, completely disappears when the RA treatment is performed after 209 hpc (black asterisk in D, E, F, L indicates the absence of Mc in the lower jaw). In parallel, the at duplicates longitudinally (P, Q, R) and, with later treatments, appeared to be triplicated (S, red asterisks indicate the duplicated at). Finally a new cartilaginous connection between the styloid process (s) and the hyoid cartilage formed as soon as proximal defect of Mc could be detected (B–E; H–K, N,O); this structure resembles closely the stylo-hyoïdo connection (sh) present in other species. No major defects where induced by RA treatments at this stages in other structures such as the sphenoid cartilage (sp) or the otic capsule (oc).

In the chondrocrania (Figures 1, 2; Figure S1), the first defects to appear were proximal malformations of Meckel's cartilage (Mc), reminiscent of the phenotype of *Dlx5* single mutants [37]. First, a medially-oriented bend appeared close to the junction between the proximal part of Mc and the malleus (Figure 1, Figure S1 and Figure 2G–L). With RA treatments performed at later times (204–210 hpc), this bend became progressively more pronounced resulting in a medially-oriented cartilage bar extending from the residual malleus towards the midline and a distal part of Mc which fused at the distal-most end of the lower jaw (Figure 1, Figure 2G–I). These distal Mc segments eventually detached from the proximal horizontal cartilage bar, became progressively smaller and made contact, through their proximo-dorsal aspect, with the ala temporalis. When RA treatment was performed between 212 and 214 hpc the Mc was reduced to its distalmost part and then disappeared (Figure 2K, L), these phenotypes resemble those observed in *Dlx5/Dlx6* combined mutant mice [39], [47] and in mice mutant for either ET1 or ETRA [46], [48]. Treatments performed at later times resulted in an almost normal development of Mc with only mild proximal defects (data not shown).

**Figure 3:**
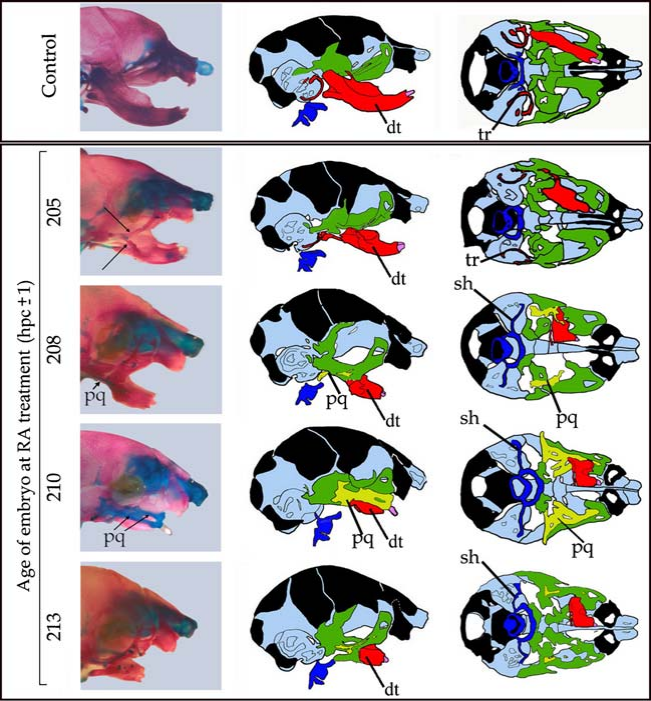
Defects in the dermatocranium induced by RA treatment. Right column represents the lateral view of representative dermatocrania obtained after RA treatment at the indicated developmental times (expressed in hpc). The second and third columns are a series of drawings representing the main features of the dermatocrania (18.5 dpc) of the same embryos. Central column, lateral view; right column, caudal view. The main structures highlighted in colour are: the dentary (dt, red), the palatoquadrate (pq, yellow), the derivatives of the maxillary component of the 1st PA (green) and the tympanic ring (tr). Note the progressive transformation of the dentary starting at 205 hpc (arrows at this stage indicate the duplicated jugal bones) accompanied by the appearance of an increasingly large palatoquadrate (pq) which, in embryos treated 210 hpc, appears as two closely anastomosed parallel bars. Note also the disappearance of the tympanic ring, the opening of the palate, the deformation of the maxillary complex and of the pterygoid bone.

During development of the middle ear in mammals, the malleus derives from the most proximal part of Mc and normally contacts the incus. The mammalian incus is considered to be the last reminiscence of the palatoquadrate, a large cartilage bar present in the upper jaw of reptilians and fish. Concomitantly with the appearance of proximal Mc defects, we observed a severe malformation of the malleus, which eventually detached from the Mc and disappeared. At the same time, the incus elongated towards the distal end of the upper jaw and contacted the proximal part of Mc with which it either formed an articulation or it anastomosed (Figure 2A–C). A cartilage condensation appeared in the center of the lateral aspect of the upper jaw, it extended progressively at both ends to occupy most of the length of the jaw contacting the defective incus with its proximal part (Figure 2D–F; M–O). This process resulted in a large, transversely flattened, bar that occupied much the same position relative to Mc and the braincase as the palatoquadrate of non-mammalian vertebrates (as in *Dlx1/2* mutants [38]) (Figure 1; Figure 2D–F, J, K; Figure S1). In the rest of this study we will refer to this structure as “palatoquadrate” (pq). In its most developed forms, the pq was constituted by two closely anastomosed parallel cartilage bars (Figure 2F; Figure 3 210 hpc), suggesting a mirror duplication (predicted in [40] for the quadruple inactivation of Dlx1/2/5/6, but never demonstrated yet), this cartilaginous structure persisted until birth.

**Figure 4:**
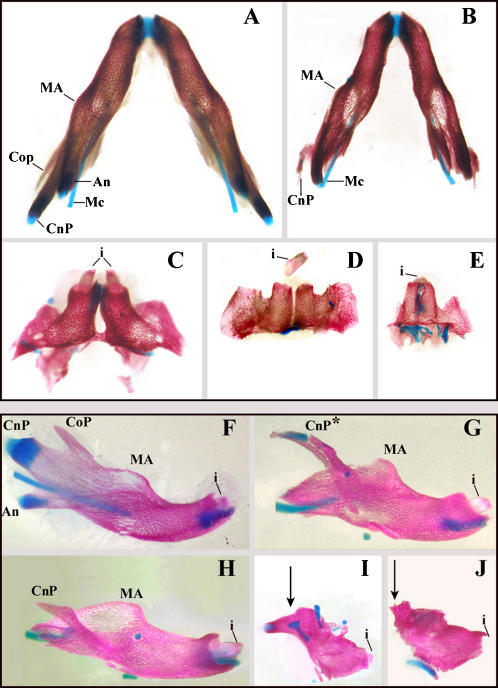
Defects of the dentary induced by RA treatment at different developmental times. Caudal (A–E) and lateral (F–J) views of dentary bones dissected from 18.5 dpc control embryos (A, F) or from embryos deriving from mothers exposed to a single dose of RA at 205 (B, G), 206 (H), 208 (C), 210 (D, I), 212 (J) and 213 (E) hpc (+/−1). The main features which can be recognized are the coronoid process (CoP), the condylar process (CnP), the angular process (An), the molar alveolus (MA) and the incisive (i). Early RA treatment results in shortening of the dentary with gradual loss of the CoP and of the An. At the same time, the CnP extends towards the upper part of the skull and contacts (or even fuses) with the jugal bone *(B,G,H*, see also Figure 6). Later RA treatments result in a dramatic reduction of the dentary which becomes a small tubular distal structure carrying an incisor. This vestigial dentary has lost all process and the MA *(C–D; I,J*); in the most severe cases the right and left remnants of the dentary fuse medially to give rise to a single, medially-located, tubular structure carrying a single incisor *(E).*

**Figure 5:**
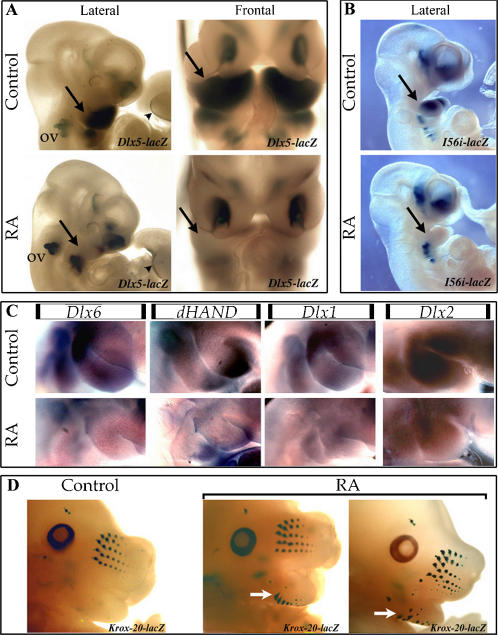
Effect of RA treatment on the specification of the maxillary and mandibular arch. *(A)* Pregnant *B6D2F1xDlx5^lacZ/+^* mice received by gavage a single dose of RA or vehicle, 207 hours after coitum. Embryos were collected 2 days later and the expression of *Dlx5-lacZ* was revealed by Xgal staining. RA treatment induced the selective down regulation of *Dlx5-lacZ* expression in the 1st PA (arrow) and did not affect its expression in other territories including the other PAs, the limb apical ectodermal ridge (arrow head), the otic vesicle (ov) and the olfactory placode. A similar result was obtained when analyzing the expression of *Dlx5* in normal embryos treated with RA at the same time (data not shown). *(B)* Transgenic mice in which the expression of *lacZ* is driven by the *Dlx5/Dlx6* intragenic enhancer *I56i*, were treated with a single dose of RA or vehicle as described above. In treated embryos collected 2 days later, expression of the reporter gene was selectively lost in the 1^st^ PA (arrow), but persisted in the brain and in the other PAs. *(C)* RA treatment of pregnant normal mice 207 hours after mating leads to a reduced expression of *Dlx6, Dlx1, Dlx2* and of *dHAND* (a target of Dlx5 and 6) in the 1^st^ PA of embryos collected at 10.5 dpc. *(D)* Transformation of the lower jaw induced by RA treatment. 14.5 dpc *Krox20-lacZ^+/−^* heterozygous embryos deriving from a mothers which had received either a single gavage of RA at 207 hpc (two representative littermates are shown, RA right panels) or vehicle alone (control: left panel) were stained with Xgal to reveal the formation of vibrissae. Note the appearance of rows of vibrissae in the lower jaw (white arrow), indicating a transformation into an upper jaw territory. The number of vibrissae in the lower jaw was highly variable even between littermates.

The ala temporalis, another maxillary derivative, was duplicated by early RA treatments (203–206 hpc) (Figure 2P–R) similarly to what is observed in *Dlx5/Dlx6* combined mutant mice; treatment at later time points resulted in the triplication of the ala temporalis (Figure 2S) which eventually disappeared with even later treatments. Starting with RA treatments at 208 hpc we observed the appearance of a cartilage structure connecting the hyoid cartilage and the styloid process (Figure 1, Figure 2N, 0). This cartilage connection, present in many vertebrates, will be referred to as the stylo-hyoid cartilage (sh). With later treatments, the sh included a lateral branching toward the larynx. RA treatments performed around 212 hpc resulted in the complete loss of most distal craniofacial components including the Mc and the pq (Figure 4; Figure 5L).

### Dynamics of dermatocranial defects induced by RA-treatment of mouse embryos

In the dermatocrania of RA-treated embryos (Figures 3, 4 and Figure S2), the dentary was progressively reduced and transformed into maxillary-like elements (Figure 4 A–E) as observed in mutants of the ET1→ETRA→Dlx5/6 pathway [39], [46]–[48]. Early RA treatments (203–205 hpc) resulted in the loss of the angular and coronoid processes and in the progressive deformation of the condylar process, which extended laterally and dorsally and contacted (and later fused) with the jugal bone of the maxillary arch (Figure 4F–H). Later RA treatments resulted in the loss of all the proximal part of the dentary including the molar and its alveolus. This profoundly deformed dentary fused dorsally with the zygomatic process of the maxilla (Figure 3; Figure 4I; Figure S2C). Furthermore, with late RA treatments, the remnants of the dentary were connected to a new bony plaque, which appeared in the upper jaw over the large cartilaginous and ramified palatoquadrate (Figure 3; Figure S2C). Late RA treatments resulted in the formation of a stylo-hyoid cartilage, which could also form a lateral branching giving rise to a laryngo-hyoidiean connection. The distalmost part of the dentary always persisted as a tubular structure, carrying an incisor, somehow reminiscent of a premaxillary bone (Fig 4D, E, J). In the most extreme cases the right and left remnants of the dentary fused medially to give a single medial tubular structure carrying a single incisor (Figure 4E). The distinct abnormal shapes of the dentary resulting from RA treatments were closely reminiscent of those observed in newborn mice in which multiple alleles of *Dlx* genes are inactivated [40].

Finally, the tympanic ring was lost coincidentally with the appearance of a pq. The palate did not close, and the pterygoids were flattened and extended medially (Figure S3).

### RA-treatment of mouse embryos leads to the progressive loss of expression of *Dlx* genes and to the transformation of the 1^st^ PA

The craniofacial defects induced by RA treatment are reminiscent of those obtained by the combinatorial inactivation of *Dlx* alleles by gene targeting in the mouse [40].

We monitored the effects of RA treatment on the expression of *Dlx* genes and their targets. At 10.5 dpc, about 48 hours after the RA pulse, the expression of *Dlx1, Dlx2*, *Dlx5, Dlx6* and the known downstream target *dHAND* [45] was strongly reduced in the 1^st ^PA (Figure 5A, C), but was not obviously affected in other structures. This effect was reminiscent of what we previously observed in *Danio rerio* [49]. The reduction of expression of *Dlx5*, *Dlx6* and *dHAND* was invariably more pronounced in the proximal part of the 1^st^ PA, while that of *Dlx1* and *Dlx2* was more evenly affected. These inhibitory effects were highly variable even among littermates (Figure S3 and data not shown).

We then analyzed the effects of RA on the activation of *I56i*, a well characterized evolutionary-conserved enhancer located in the *Dlx5-6* intergenic region [50]. This enhancer is involved in ET1 signal transduction (Kurihara, unpublished observations). Treatment of pregnant *I56i-lacZ* mice with RA in the critical period described above, specifically inhibited the enhancer activity in the 1^st^ PA leaving it unaffected in the brain and in other PAs (Figure 5B and Figure S3) supporting the notion that RA treatment affects the ET1-mediated activation of *Dlx5/6* through enhancer *I56i*.

The combined inactivation of *Dlx5* and *Dlx6* in the mouse leads to a lower-to-upper jaw transformation, with vibrissae forming also in the lower jaw [39], [40]. In order to see if a similar transformation was taking place following the reduced expression of *Dlx5* and *6* in the 1^st^ PA of RA-treated embryos we analyzed the effects of RA on *Krox20-lacZ^+/−^* mice, in which the *lacZ* reporter is expressed by the developing vibrissae [51]. When pregnant *Krox20-lacZ^+/−^* mice were treated with RA, vibrissae pads appeared ectopically in the lower jaw of 14.5 dpc embryos (6 days after treatment) (Figure 5D) indicating that the lower-to upper jaw transformation had indeed taken place.

### RA treatment leads to a reduction of the territories of Fgf8 and ET1 expression in the epithelium of the 1^st^ PA

**Figure 6:**
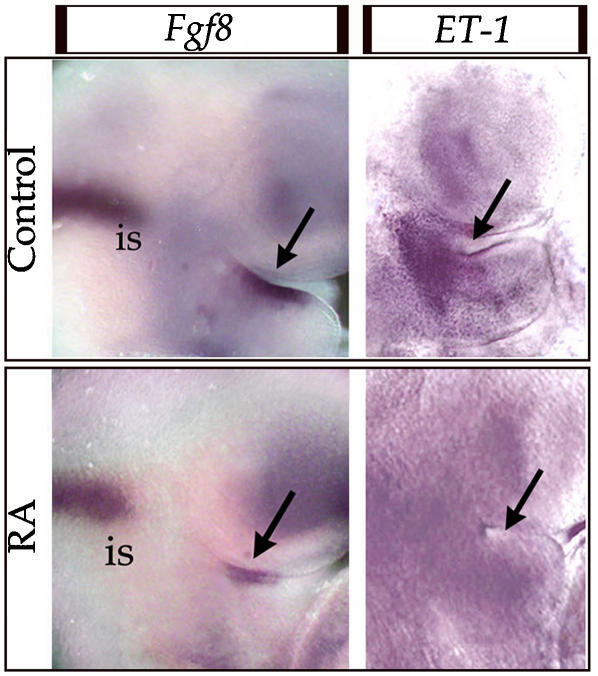
Reduction of the territories of expression of Fgf8 and ET1 induced by RA treatment in the 1^st^ PA. *In situ* hybridization with an *Fgf8* and an *ET1* probe on the head region of normal embryos collected 4h after treating the mother with vehicle or RA at 207 hpc. Note that the territory of expression of both genes in the signalling territory of the epithelium of the 1^st^ PA (black arrow) is strongly reduced, while the level of expression of Fgf8 in the isthmus (is) was not significantly affected by RA treatment.

In the 1^st^ PA *Dlx* genes are mostly expressed by the mesenchyme derived from CNCCs. Given the timing of treatment and the absence of RARs in CNCCs, the reduced expression of *Dlx* genes could result from an altered production of their known inducing molecules by the 1^st^ PA signalling centers: ET1 and Fgf8. We analyzed embryos 4 hours after RA treatment, and we observed a clear reduction in the territory of *ET1* and *Fgf8* expression within the 1^st^ PA epithelium (Figure 6). Notably this effect appears to be specific for the 1^st^ PA, as the expression of *Fgf8* in the isthmus of the same embryos was unaffected by exposure to RA.

## Discussion

In this study, we provide an explanation for the origin of craniofacial malformations induced by RA exposure. We show that one single RA treatment of pregnant mice carrying embryos of 8.5–8.75 dpc results in a series of craniofacial defects affecting most of the 1^st^ PA. The developmental stage at which RA treatment of the mother results in craniofacial malformations is 204 to 213 hpc i.e. embryos of 9 to 14 somites. This is the window of time during which CNCCs from rhombomeres 1 and 2 and the mesencephalon reach the 1^st^ PA [11]. When analyzing the chondrocrania or the dermatocrania of treated embryos, we observe that the defects deriving from the earlier treatments affect only proximal derivatives of the 1^st^ PA such as the proximal part of Mc or the malleus, while treatments performed at later times affect both proximal and distal structures. These morphological defects are mirrored by the progressive reduction of the territories of *Dlx* gene expression which is first reduced only in proximal parts of the 1^st^ PA and then progressively lost from most of the 1^st^ PA, persisting always only in the distalmost region. Most of the defects induced by RA treatment can be interpreted as the direct consequence of the progressive reduction in the level of expression of *Dlx* genes. Indeed, similar defects are observed either in mice in which *Dlx* genes have been inactivated [37]–[40] or in mice carrying different *Dlx* allelic combinations obtained by cross breeding different mutants [40].

**Figure 7:**
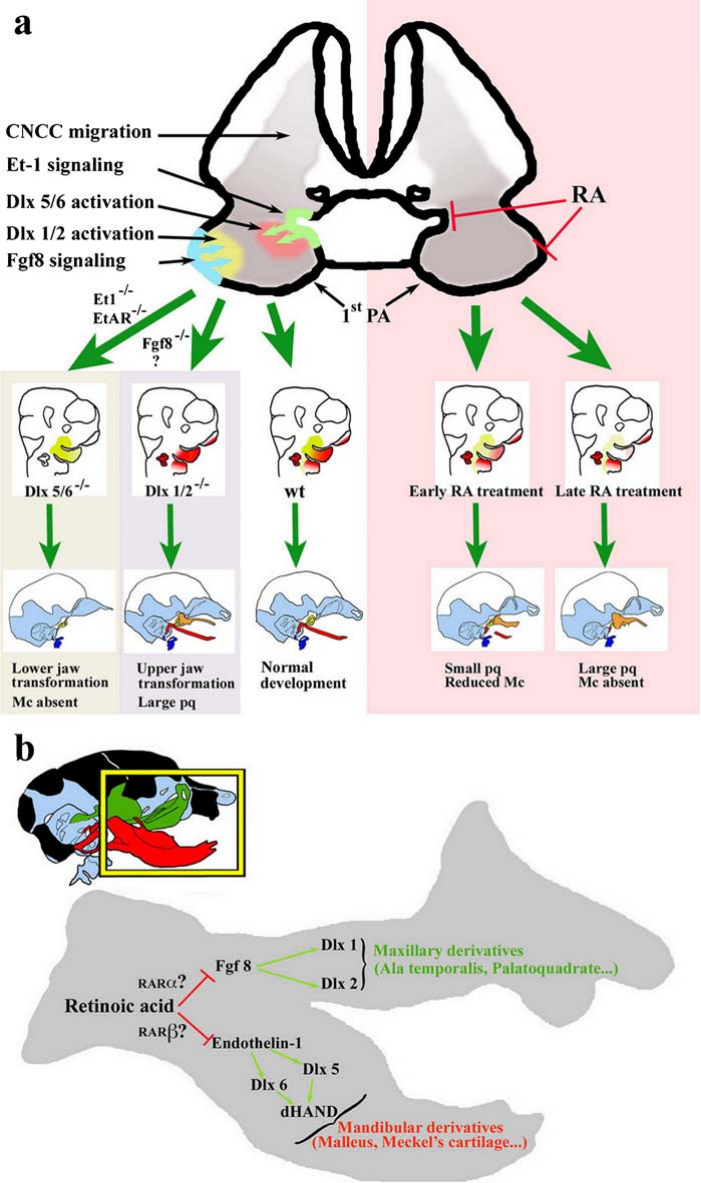
Summary diagram of the effects of RA on derivatives of the 1^st^ PA. *(A)* RA treatment (right side) inhibits both Fgf8 (blue) and ET1 (green) signalling from the 1^st^ PA epithelium and gradually prevents the activation of *Dlx1/Dlx2* (yellow) and *Dlx5/Dlx6* (red) in the CNCC-derived ectomesenchyme. This results in phenotypic combinations of altered characters reminiscent of those present in the *Dlx1/2* and *Dlx5/6* double mutants. *(B)* Summary diagram of the effects of RA-treatment during jaw development.

In the 1^st^ PA *Dlx* genes are expressed by incoming CNCCs only in response to molecular signals emanating from the PA epithelial lining. *Dlx5* and *Dlx6* are activated by ET1 secretion from the pharyngeal endoderm [45], [46]. Inactivation of either ET1 or its receptor A completely inhibit their expression in the underlying ectomesenchyme [46], [48]. *Dlx1* and *Dlx2* are activated in the ectomesenchyme by Fgf8 secreted from the epithelium of the 1^st^ PA [42], [43]. Our results indicate that RA-mediated repression of *Dlx* genes in CNCCs is not direct. First, we show that after RA-treatment, *Dlx* genes are selectively downregulated in the 1^st^ PA, but not in other PAs or in other structures such as the otic vesicle or the limbs. This suggests that this regulation does not reflect a generalized effect of RA. Second, concerning more specifically *Dlx5* and *Dlx6* we show that their RA-repression is mediated through *I56i* a well characterized enhancer which does not contain retinoic acid responsive elements [50] and which is activated by ET1. The defective expression of *Dlx* genes in the mesenchyme of the 1^st^ PA and the morphological effects of RA treatment could derive from the reduction Fgf8 and ET1 signalling from the 1^st^ PA epithelium to CNCCs. However, in the absence of direct rescue experiments, excessively difficult in the early mouse embryo, the contribution of ET1 and Fgf8 pathways on RA-induced craniofacial malformations remains still to be directly demonstrated. To circumvent this difficulty we are reproducing a similar analysis on other vertebrate species (chick, Xenopus) in which localized rescue experiments can be designed more easily. In any event, the ET1→ETRA→Dlx5/6 and the Fgf8→Dlx1/2 pathways do not seem to be mutually interacting as their genetic perturbation by gene targeting in the mouse leads to distinct and non overlapping phenotypes: the first results in the transformation of the lower jaw in an upper jaw, the second in the appearance of a pq (summarized in Figure 7).

As mentioned in the introduction, two further sets of data reinforce the notion that the lesion induced by RA in the specific window of time we have chosen, depends on altered signalling from the pharyngeal epithelia. First of all grafting experiments in the chick have demonstrated the critical importance of endodermal and ectodermal signalling for patterning CNCCs [29], [30]. Second, recent findings show that treatment of mouse embryos with a general agonist of RA, activates ectopically RA signalling in the endoderm and ectoderm lining the first two PAs [22]. In this same study it is clearly shown that RA cannot elicit a RA-receptor mediated response in CNCC at this stage of development.

Many experimental indications support the notion that the teratogenic effects of retinoids are receptor-mediated. First of all, those synthetic retinoids which are incapable to activate retinoic acid receptors (RARs) are not teratogenic [52], whereas administration of agonists for different RAR isotypes results in isotype-specific congenital defects [53], [54]. Second, mouse embryos lacking either RARγ or RXRα are resistant to many RA-induced malformations [55]–[58], whereas inactivation of *RARγ* restores the viability of mice lacking the RA-degrading enzyme CYP26A1 [59]. The physiological roles of endogenous RA in the control of developmental processes do not seem to be generally reflected by the teratogenic effects resulting from administration of exogenous RA to embryos. Indeed, RA can lead to teratogenic effects at stages where RA is not normally synthesized in the embryo [60] and the spectrum of malformations induced by excess of RA are different from those observed in mice in which RA signalling has been genetically or pharmacologically impaired [61]–[64].

RA-dependent inactivation of the ET1 pathway might involve RARß/RXR heterodimers, which are induced in the endoderm and are known to act as ET1 suppressors [22], [65]; inactivation of Fgf8 expression could involve RARα [66].

Our results reinforce the notion that the exact topography and timing of epithelial signalling to CNCC is essential to determine different craniofacial morphotypes [42]. In particular, our findings imply that for normal craniofacial development of the mouse it is essential to protect specific signalling regions of the 1^st^ PA from the action of RA. Remarkably, the RA-degrading enzymes CYP26A1 and CYP26C1 are expressed in these regions when CNCCs reach the 1^st^ PA [67], [68].

Depending on the exact age of the embryo at treatment, we have observed the co-variance of several craniofacial characters leading to morphologies that somewhat recall the primitive condition of jawed vertebrate skulls where the palatoquadrate is separate from the braincase. A particularly vivid example can be seen at the level of the inner ear. The progressive transformation of the incus into a pq (caused by the loss of Dlx1/2) is paralleled by that of the malleus into the proximal part of Mc (caused by the loss of Dlx5/6). These two simultaneous transformations generate an articulation between the proximal part of the transformed Mc and the pq. This articulation is a typical character of primitive jaws, which, in modern gnathostomes, persists between the malleus and the incus. Furthermore the co-variance of other characters, such as the appearance of a sh connection can be regarded as an example of how mutations affecting the regulation of an early morphogenetic process can lead to the co-evolution of many characters of the same organism, that are presumed not to be functionally related.

Similar mechanisms might have been more generally involved in determining the development and evolution of animal form and could account for what is often depicted as bursts of anatomical innovations in evolutionary scenarios.

## Methods

### Experimental Procedures

#### Mice

Normal B6D2F1 hybrid mice (Charles River Laboratory, France) 9 to 28 weeks old were used throughout this study. Generation and genotyping of the *Dlx5^LacZ/+^*, *Dlx5/Dlx6^neo/+^, Krox20^LacZ/+^, Et1^neo/+^* have been previously described [37], [39], [51], [69], these mutations were maintained on the B6D2F1 hybrid genetic background. Lines of transgenic mice expressing *lacZ* under the control of the mouse *I56i* enhancer were previously described [50]. For timed mating, male and female mice were placed in the same cage at 7 pm (1 hour before light extinction), vaginal plugs were detected at 9 pm and the mice were then separated, 8 pm was considered as the average mating hour. For non-timed mating, mice were left together through the night and vaginal plugs were detected at 9 am next morning.

#### RA administration

We treated mouse embryos by gavage of the mother with RA at precise times after mating. This approach, largely used to study the effects of RA on axial skeletal development, has been difficult to apply to the analysis of craniofacial abnormalities due to complex and variable phenotypes observed even among littermates. To chose the window of time most appropriate to study craniofacial malformations we made reference to previous reports. Treatments of mouse embryos between 7 and 8 dpc are known to result in severe axial defects including vertebral and CNS transformations [70], [71] with no major effects observed on craniofacial structures. Most craniofacial effects have been observed with treatments performed shortly after this period [71], [72], however the defects observed were very variable and a detailed description of the lesions is still missing. To better control the time of RA exposure, we systematically administered a single dose of RA at times after mating ranging from 200 to 224 hours post coitum (hpc). As after oral administration RA is quickly degraded [73], this protocol results in exposing embryos to pharmacological levels of RA for less than 2 hours. We then analyzed the effects of this early RA exposure in 10.5, 14.5 and 18.5 dpc embryos, therefore approximately 2, 6 and 10 days after treatment.

A solution of all-trans-retinoic acid (Sigma, France) (25 mg/ml in dimethylsulphoxide) was diluted l/10 in olive oil just before use, and about 0.3 ml were delivered by a single gavage for a final dose of 25 mg/Kg of maternal body weight. The gavage was performed on the 8th dpc at times varying between 6 am and 9 pm. Control mice received the same mixture without RA. To collect embryos at 10.5, 14.5 and 18.5 dpc pregnant mice were anesthetized and sacrificed by cervical dislocation at 12 am of the corresponding day. To detect the direct effect of RA on gene expression of *Fgf8* and *ET-1* mice embryos were collected 4h after gavage.

#### Whole-mount RNA *in situ* hybridization

RNA *in situ* hybridation was performed using a digoxigenin (DIG)-UTP (Roche) labelled antisense RNA probe. Signal was detected using an alkaline-phosphatase-conjugated anti-DIG antibody and NBT/BCIP substrate (Roche). Whole mount hybridisation was carried out as described [46] with slight modifications.

The *dHand* probe corresponded to 450 bp in the 3′ end of *dHand* cDNA and was linearized with EcoRI and transcribed with T7 RNA polymerase. The *Dlx1* probe comprised 720 bp of the 3′ end of murine *Dlx1* cDNA and was linearized with BamHI and transcribed with T7 RNA polymerase. The *Dlx5* probe comprised 780bp and was linearized with EcoRI and transcribed with T7 RNA polymerase. The *Dlx6* probe comprised 370bp and was linearized with SpeI and transcribed with T7 RNA polymerase. The *Et1* probe corresponded to 1000bp of mouse *Et1* cDNA ORF and was linearized with ApaI and transcribed with Sp6 RNA polymerase. The *Fgf8* probe comprised 800bp of the 3′ UTR and the entire coding region of a spliced variant of mouse Fgf8; it was linearized with PstI and transcribed with T7 RNA polymerase.

#### Skeletal Preparations

Cartilage staining of E14.5 embryos as well as bone and cartilage staining of E18.5 embryos were carried out as previously described [37].

#### β-galactosidase staining

For *lacZ* expression analysis 10.5 dpc embryos were fixed for 15–30 min in 2% paraformaldehyde (PFA) in PBS, while 14.5 dpc embryos were fixed for 15–30 min in 4% PFA. Xgal staining was performed as described [37].

## Supporting Information

Figure S1 Chondrocranial defects induced by RA. Lateral (right) and caudal (left) views of the chondrocrania of representatice 14.5 dpc embryos stained with alcian blue. The embryos derive from litters obtained after treatment of the pregnant mother at the indicated developmental time (expressed in hours post coitum). Black arrow: Meckel's cartilage, red arrow: palatoquadrate.
(8.01 MB TIF)

Figure S2 Defects in the base of the dermatocranium induced by RA treatment. Images of the base of the skull of skeletal preparations from a 18.5 dpc control embryo (A) or from embryos deriving from mothers exposed to a single dose of RA at 205 (B) or 210 (C) hpc. With early RA treatments (B), the proximal part of the dt is lost, later treatments (C) result is a dramatic reduction of the dt which is reduced to a small distal part which is connected to a new bony plaque which appears in the upper jaw over the large cartilagineous palatoquadrate (pq). RA treatments result in the formation of a stylo-hyoidiean connection (sh), which, with later treatments, forms also a lateral branching giving rise to a laryngo-hyoidiean connection (Lh). Abbreviations: dt: dentary, tr, tympanic ring; hy, hyoid bone; oc, otic capsule, sh stylo-hyoidiean connection; Lh, laryngo-hyoidiean connection.
(6.53 MB TIF)

Figure S3 Variability of RA effects on gene expression in the 1st PA even between littermates. I56i-lacZ embryos at 10.5 dpc deriving from a litter treated with a single pulse of RA at 207 hpc. In all cases we observe a reduction of the territory of expression of the reporter gene in the 1st PA (arrow), but not in other positive structures such as the brain (br) or other PAs. On the right, a control embryo issued from an untreated litter.
(4.28 MB TIF)

## References

[1] Soprano DR, Soprano KJ (1995). Retinoids as teratogens.. Annu Rev Nutr.

[2] Collins MD, Mao GE (1999). Teratology of retinoids.. Annu Rev Pharmacol Toxicol.

[3] Escriva H, Holland ND, Gronemeyer H, Laudet V, Holland LZ (2002). The retinoic acid signaling pathway regulates anterior/posterior patterning in the nerve cord and pharynx of amphioxus, a chordate lacking neural crest.. Development.

[4] Lammer EJ, Chen DT, Hoar RM, Agnish ND, Benke PJ (1985). Retinoic acid embryopathy.. N Engl J Med.

[5] Mark M, Chambon P (2003). Functions of RARs and RXRs in vivo: Genetic dissection of the retinoid signaling pathway.. Pure and applied chemistry.

[6] Mallo M (1997). Retinoic acid disturbs mouse middle ear development in a stage-dependent fashion.. Dev Biol.

[7] Coberly S, Lammer E, Alashari M (1996). Retinoic acid embryopathy: case report and review of literature.. Pediatr Pathol Lab Med.

[8] Creuzet S, Couly G, Le Douarin NM (2005). Patterning the neural crest derivatives during development of the vertebrate head: insights from avian studies.. J Anat.

[9] Santagati F, Rijli FM (2003). Cranial neural crest and the building of the vertebrate head.. Nat Rev Neurosci.

[10] Kontges G, Lumsden A (1996). Rhombencephalic neural crest segmentation is preserved throughout craniofacial ontogeny.. Development.

[11] Serbedzija GN, Bronner-Fraser M, Fraser SE (1992). Vital dye analysis of cranial neural crest cell migration in the mouse embryo.. Development.

[12] Cheung CS, Wang L, Dong M, Chan WY (2003). Migration of Hindbrain Neural Crest Cells in the Mouse.. neuroembryology.

[13] Trainor PA, Melton KR, Manzanares M (2003). Origins and plasticity of neural crest cells and their roles in jaw and craniofacial evolution.. Int J Dev Biol.

[14] Lumsden A, Sprawson N, Graham A (1991). Segmental origin and migration of neural crest cells in the hindbrain region of the chick embryo.. Development.

[15] Goulding EH, Pratt RM (1986). Isotretinoin teratogenicity in mouse whole embryo culture.. J Craniofac Genet Dev Biol.

[16] Lee YM, Osumi-Yamashita N, Ninomiya Y, Moon CK, Eriksson U (1995). Retinoic acid stage-dependently alters the migration pattern and identity of hindbrain neural crest cells.. Development.

[17] Pratt RM, Goulding EH, Abbott BD (1987). Retinoic acid inhibits migration of cranial neural crest cells in the cultured mouse embryo.. J Craniofac Genet Dev Biol.

[18] Webster WS, Johnston MC, Lammer EJ, Sulik KK (1986). Isotretinoin embryopathy and the cranial neural crest: an in vivo and in vitro study.. J Craniofac Genet Dev Biol.

[19] Wei X, Makori N, Peterson PE, Hummler H, Hendrickx AG (1999). Pathogenesis of retinoic acid-induced ear malformations in primate model.. Teratology.

[20] Alles AJ, Sulik KK (1992). Pathogenesis of retinoid-induced hindbrain malformations in an experimental model.. Clin Dysmorphol.

[21] Sulik KK, Cook CS, Webster WS (1988). Teratogens and craniofacial malformations: relationships to cell death.. Development.

[22] Matt N, Ghyselinck NB, Wendling O, Chambon P, Mark M (2003). Retinoic acid-induced developmental defects are mediated by RARbeta/RXR heterodimers in the pharyngeal endoderm.. Development.

[23] Ruberte E, Dolle P, Chambon P, Morriss-Kay G (1991). Retinoic acid receptors and cellular retinoid binding proteins. II. Their differential pattern of transcription during early morphogenesis in mouse embryos.. Development.

[24] Dolle P, Ruberte E, Leroy P, Morriss-Kay G, Chambon P (1990). Retinoic acid receptors and cellular retinoid binding proteins. I. A systematic study of their differential pattern of transcription during mouse organogenesis.. Development.

[25] Iulianella A, Lohnes D (2002). Chimeric analysis of retinoic acid receptor function during cardiac looping.. Dev Biol.

[26] Dupe V, Ghyselinck NB, Wendling O, Chambon P, Mark M (1999). Key roles of retinoic acid receptors alpha and beta in the patterning of the caudal hindbrain, pharyngeal arches and otocyst in the mouse.. Development.

[27] Jiang X, Choudhary B, Merki E, Chien KR, Maxson RE (2002). Normal fate and altered function of the cardiac neural crest cell lineage in retinoic acid receptor mutant embryos.. Mech Dev.

[28] Wendling O, Dennefeld C, Chambon P, Mark M (2000). Retinoid signaling is essential for patterning the endoderm of the third and fourth pharyngeal arches.. Development.

[29] Haworth KE, Healy C, Morgan P, Sharpe PT (2004). Regionalisation of early head ectoderm is regulated by endoderm and prepatterns the orofacial epithelium.. Development.

[30] Couly G, Creuzet S, Bennaceur S, Vincent C, Le Douarin NM (2002). Interactions between Hox-negative cephalic neural crest cells and the foregut endoderm in patterning the facial skeleton in the vertebrate head.. Development.

[31] Manley NR, Capecchi MR (1998). Hox group 3 paralogs regulate the development and migration of the thymus, thyroid, and parathyroid glands.. Dev Biol.

[32] Mulder GB, Manley N, Maggio-Price L (1998). Retinoic acid-induced thymic abnormalities in the mouse are associated with altered pharyngeal morphology, thymocyte maturation defects, and altered expression of Hoxa3 and Pax1.. Teratology.

[33] Gavalas A, Trainor P, Ariza-McNaughton L, Krumlauf R (2001). Synergy between Hoxa1 and Hoxb1: the relationship between arch patterning and the generation of cranial neural crest.. Development.

[34] Graham A, Smith A (2001). Patterning the pharyngeal arches.. Bioessays.

[35] Veitch E, Begbie J, Schilling TF, Smith MM, Graham A (1999). Pharyngeal arch patterning in the absence of neural crest.. Curr Biol.

[36] Couly G, Grapin-Botton A, Coltey P, Ruhin B, Le Douarin NM (1998). Determination of the identity of the derivatives of the cephalic neural crest: incompatibility between Hox gene expression and lower jaw development.. Development.

[37] Acampora D, Merlo GR, Paleari L, Zerega B, Postiglione MP (1999). Craniofacial, vestibular and bone defects in mice lacking the Distal-less-related gene Dlx5.. Development.

[38] Qiu M, Bulfone A, Ghattas I, Meneses JJ, Christensen L (1997). Role of the Dlx homeobox genes in proximodistal patterning of the branchial arches: mutations of Dlx-1, Dlx-2, and Dlx-1 and -2 alter morphogenesis of proximal skeletal and soft tissue structures derived from the first and second arches.. Dev Biol.

[39] Beverdam A, Merlo GR, Paleari L, Mantero S, Genova F (2002). Jaw transformation with gain of symmetry after Dlx5/Dlx6 inactivation: mirror of the past?. Genesis.

[40] Depew MJ, Simpson CA, Morasso M, Rubenstein JL (2005). Reassessing the Dlx code: the genetic regulation of branchial arch skeletal pattern and development.. J Anat.

[41] Shigetani Y, Nobusada Y, Kuratani S (2000). Ectodermally derived FGF8 defines the maxillomandibular region in the early chick embryo: epithelial-mesenchymal interactions in the specification of the craniofacial ectomesenchyme.. Dev Biol.

[42] Shigetani Y, Sugahara F, Kawakami Y, Murakami Y, Hirano S (2002). Heterotopic shift of epithelial-mesenchymal interactions in vertebrate jaw evolution.. Science.

[43] Park BK, Sperber SM, Choudhury A, Ghanem N, Hatch GT (2004). Intergenic enhancers with distinct activities regulate Dlx gene expression in the mesenchyme of the branchial arches.. Dev Biol.

[44] Chai Y, Maxson RE (2006). Recent advances in craniofacial morphogenesis.. Dev Dyn.

[45] Charite J, McFadden DG, Merlo G, Levi G, Clouthier DE (2001). Role of Dlx6 in regulation of an endothelin-1-dependent, dHAND branchial arch enhancer.. Genes Dev.

[46] Ruest LB, Xiang X, Lim KC, Levi G, Clouthier DE (2004). Endothelin-A receptor-dependent and -independent signaling pathways in establishing mandibular identity.. Development.

[47] Depew MJ, Lufkin T, Rubenstein JL (2002). Specification of jaw subdivisions by Dlx genes.. Science.

[48] Ozeki H, Kurihara Y, Tonami K, Watatani S, Kurihara H (2004). Endothelin-1 regulates the dorsoventral branchial arch patterning in mice.. Mech Dev.

[49] Ellies DL, Langille RM, Martin CC, Akimenko MA, Ekker M (1997). Specific craniofacial cartilage dysmorphogenesis coincides with a loss of dlx gene expression in retinoic acid-treated zebrafish embryos.. Mech Dev.

[50] Zerucha T, Stuhmer T, Hatch G, Park BK, Long Q (2000). A highly conserved enhancer in the Dlx5/Dlx6 intergenic region is the site of cross-regulatory interactions between Dlx genes in the embryonic forebrain.. J Neurosci.

[51] Schneider-Maunoury S, Topilko P, Seitandou T, Levi G, Cohen-Tannoudji M (1993). Disruption of Krox-20 results in alteration of rhombomeres 3 and 5 in the developing hindbrain.. Cell.

[52] Kochhar DM, Jiang H, Penner JD, Beard RL, ChandraratnaAs (1996). Differential teratogenic response of mouse embryos to receptor selective analogs of retinoic acid.. Chem Biol Interact.

[53] Arafa HM, Elmazar MM, Hamada FM, Reichert U, Shroot B (2000). Selective agonists of retinoic acid receptors: comparative toxicokinetics and embryonic exposure.. Arch Toxicol.

[54] Elmazar MM, Reichert U, Shroot B, Nau H (1996). Pattern of retinoid-induced teratogenic effects: possible relationship with relative selectivity for nuclear retinoid receptors RAR alpha, RAR beta, and RAR gamma.. Teratology.

[55] Nugent P, Sucov HM, Pisano MM, Greene RM (1999). The role of RXR-alpha in retinoic acid-induced cleft palate as assessed with the RXR-alpha knockout mouse.. Int J Dev Biol.

[56] Sucov HM, Izpisua-Belmonte JC, Ganan Y, Evans RM (1995). Mouse embryos lacking RXR alpha are resistant to retinoic-acid-induced limb defects.. Development.

[57] Iulianella A, Lohnes D (1997). Contribution of retinoic acid receptor gamma to retinoid-induced craniofacial and axial defects.. Dev Dyn.

[58] Lohnes D, Mark M, Mendelsohn C, Dolle P, Dierich A (1994). Function of the retinoic acid receptors (RARs) during development (I). Craniofacial and skeletal abnormalities in RAR double mutants.. Development.

[59] Abu-Abed S, Dolle P, Metzger D, Wood C, MacLean G (2003). Developing with lethal RA levels: genetic ablation of Rarg can restore the viability of mice lacking Cyp26a1.. Development.

[60] Sulik KK, Dehart DB, Rogers JM, Chernoff N (1995). Teratogenicity of low doses of all-trans retinoic acid in presomite mouse embryos.. Teratology.

[61] Elmazar MM, Ruhl R, Nau H (2001). Synergistic teratogenic effects induced by retinoids in mice by coadministration of a RARalpha-or RARgamma-selective agonist with a RXR-selective agonist.. Toxicol Appl Pharmacol.

[62] Elmazar MM, Nau H (2004). Potentiation of the teratogenic effects induced by coadministration of retinoic acid or phytanic acid/phytol with synthetic retinoid receptor ligands.. Arch Toxicol.

[63] Alles AJ, Sulik KK (1990). Retinoic acid-induced spina bifida: evidence for a pathogenetic mechanism.. Development.

[64] Knudsen PA (1967). Dental malformations in rat embryos with exencephaly induced by hypervitaminosis A.. Acta Odontol Scand.

[65] Yokota J, Kawana M, Hidai C, Aoka Y, Ichikawa K (2001). Retinoic acid suppresses endothelin-1 gene expression at the transcription level in endothelial cells.. Atherosclerosis.

[66] Brondani V, Klimkait T, Egly JM, Hamy F (2002). Promoter of FGF8 reveals a unique regulation by unliganded RARalpha.. J Mol Biol.

[67] Tahayato A, Dolle P, Petkovich M (2003). Cyp26C1 encodes a novel retinoic acid-metabolizing enzyme expressed in the hindbrain, inner ear, first branchial arch and tooth buds during murine development.. Gene Expr Patterns.

[68] Sakai Y, Meno C, Fujii H, Nishino J, Shiratori H (2001). The retinoic acid-inactivating enzyme CYP26 is essential for establishing an uneven distribution of retinoic acid along the anterio-posterior axis within the mouse embryo.. Genes Dev.

[69] Kurihara Y, Kurihara H, Suzuki H, Kodama T, Maemura K (1994). Elevated blood pressure and craniofacial abnormalities in mice deficient in endothelin-1.. Nature.

[70] Kessel M, Gruss P (1991). Homeotic transformations of murine vertebrae and concomitant alteration of Hox codes induced by retinoic acid.. Cell.

[71] Simeone A, Avantaggiato V, Moroni MC, Mavilio F, Arra C (1995). Retinoic acid induces stage-specific antero-posterior transformation of rostral central nervous system.. Mech Dev.

[72] Morriss-Kay G, Ruberte E, Fukiishi Y (1993). Mammalian neural crest and neural crest derivatives.. Ann Anat.

[73] Saadeddin A, Torres-Molina F, Carcel-Trullols J, Araico A, Peris JE (2004). Pharmacokinetics of the time-dependent elimination of all-trans-retinoic acid in rats.. AAPS PharmSci.

