# Supplementary figures and images for: Molecular Dynamics of Retinoic Acid-Induced Craniofacial Malformations: Implications for the Origin of Gnathostome Jaws
Source: PLoS One. 2007 Jun 6;2(6):e510. doi: 10.1371/journal.pone.0000510 (PMC1876820; doi:10.1371/journal.pone.0000510)

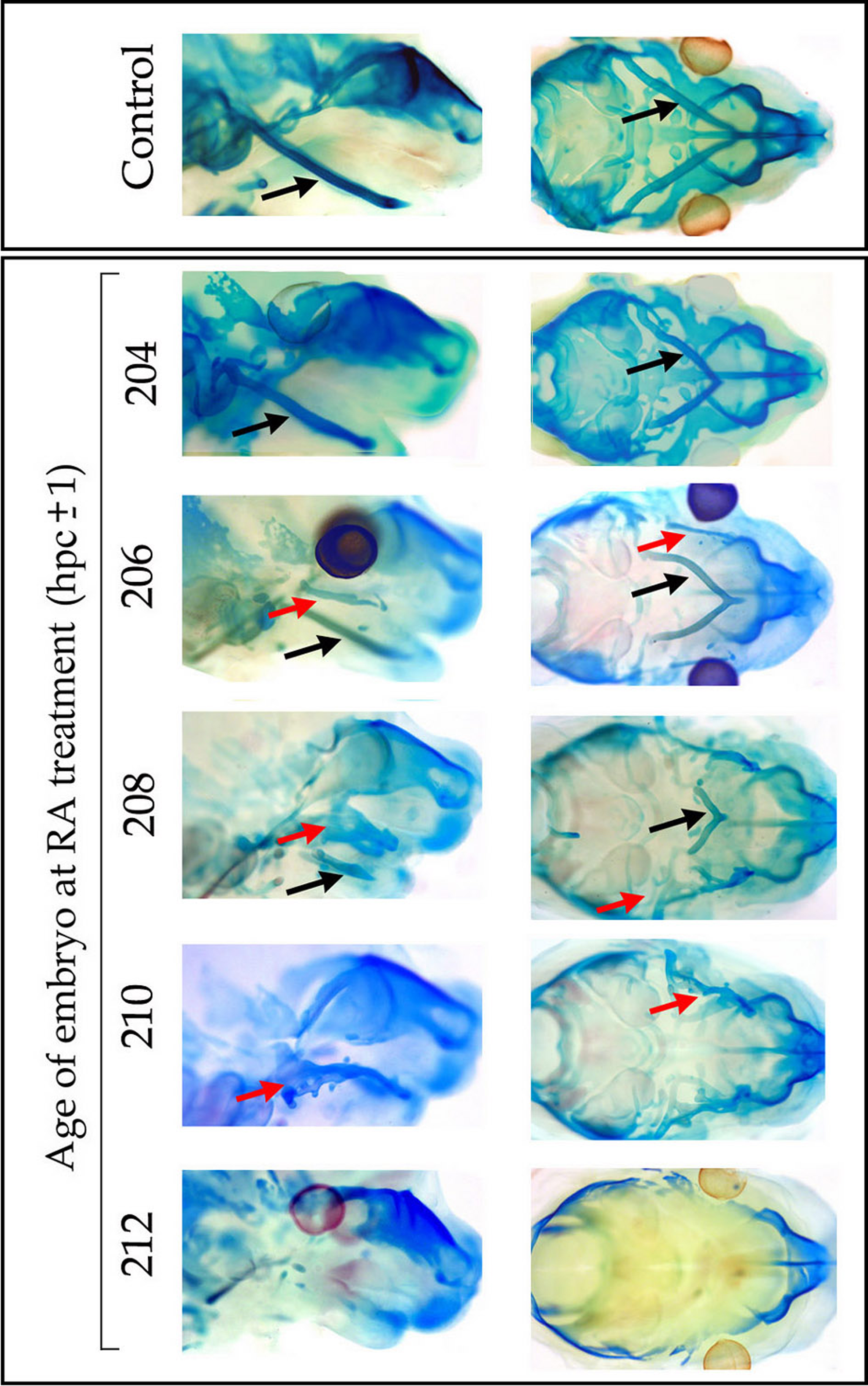

Supplement: Figure S1 — Chondrocranial defects induced by RA. Lateral (right) and caudal (left) views of the chondrocrania of representatice 14.5 dpc embryos stained with alcian blue. The embryos derive from litters obtained after treatment of the pregnant mother at the indicated developmental time (expressed in hours post coitum). Black arrow: Meckel's cartilage, red arrow: palatoquadrate. (8.01 MB TIF) [file pone.0000510.s001.tif]

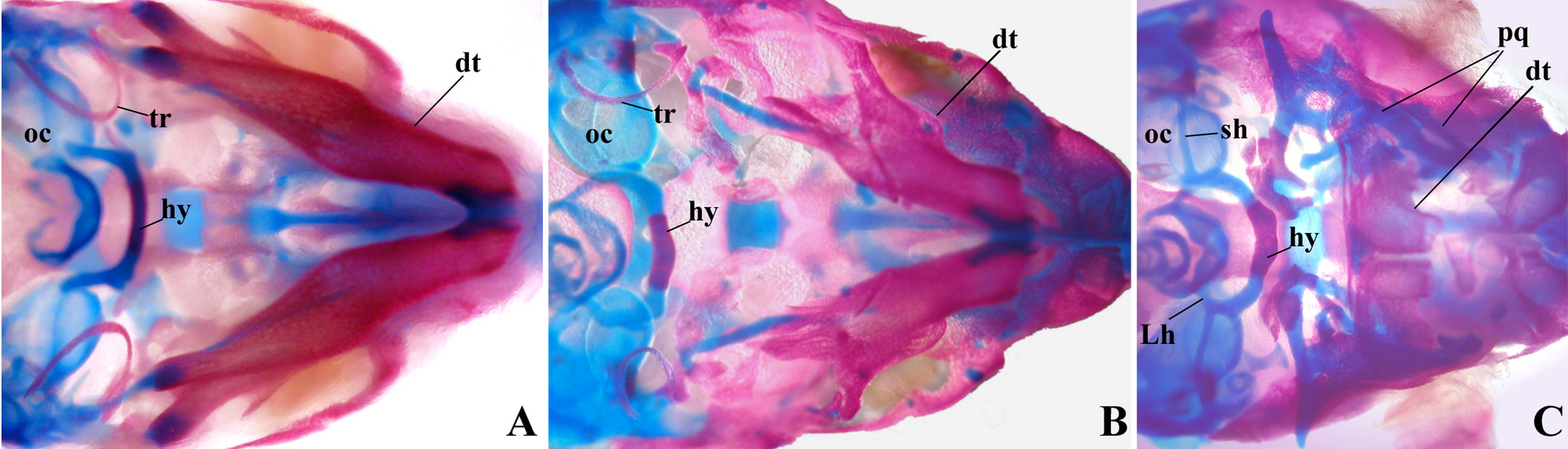

Supplement: Figure S2 — Defects in the base of the dermatocranium induced by RA treatment. Images of the base of the skull of skeletal preparations from a 18.5 dpc control embryo (A) or from embryos deriving from mothers exposed to a single dose of RA at 205 (B) or 210 (C) hpc. With early RA treatments (B), the proximal part of the dt is lost, later treatments (C) result is a dramatic reduction of the dt which is reduced to a small distal part which is connected to a new bony plaque which appears in the upper jaw over the large cartilagineous palatoquadrate (pq). RA treatments result in the formation of a stylo-hyoidiean connection (sh), which, with later treatments, forms also a lateral branching giving rise to a laryngo-hyoidiean connection (Lh). Abbreviations: dt: dentary, tr, tympanic ring; hy, hyoid bone; oc, otic capsule, sh stylo-hyoidiean connection; Lh, laryngo-hyoidiean connection. (6.53 MB TIF) [file pone.0000510.s002.tif]

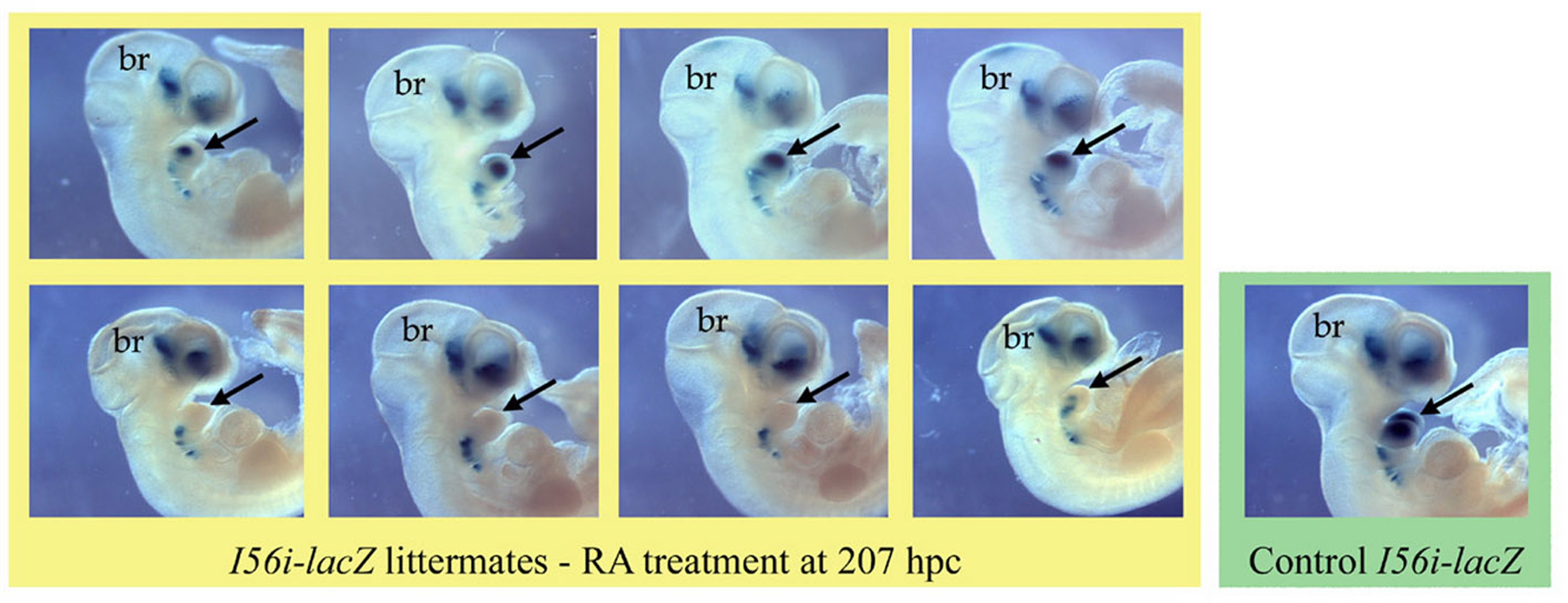

Supplement: Figure S3 — Variability of RA effects on gene expression in the 1st PA even between littermates. I56i-lacZ embryos at 10.5 dpc deriving from a litter treated with a single pulse of RA at 207 hpc. In all cases we observe a reduction of the territory of expression of the reporter gene in the 1st PA (arrow), but not in other positive structures such as the brain (br) or other PAs. On the right, a control embryo issued from an untreated litter. (4.28 MB TIF) [file pone.0000510.s003.tif]
